# Long‐term ecological data for conservation: Range change in the black‐billed capercaillie (*Tetrao urogalloides*) in northeast China (1970s–2070s)

**DOI:** 10.1002/ece3.3859

**Published:** 2018-03-23

**Authors:** Li Yang, Chao Zhang, Minhao Chen, Jingxin Li, Lei Yang, Zhaomin Huo, Shahid Ahmad, Xiaofeng Luan

**Affiliations:** ^1^ School of Nature Conservation Beijing Forestry University Beijing China

**Keywords:** biogeography, black‐billed capercaillie, climate change, long‐term ecological data, *Tetrao urogalloides*

## Abstract

Long‐term ecological data can be an effective tool to help ecologists integrate future projections with historical contexts and provide unique insights into the long‐term dynamics of endangered species. However, hampered by data limitations, including incomplete and spatially biased data, relatively few studies have used multidecadal datasets or have examined changes in biogeography from a historical perspective. The black‐billed capercaillie (*Tetrao urogalloides*) is a large capercaillie (classified as Least Concern [LC] on the IUCN red list) that has undergone a dramatic decline in population during the late 20th century and is considered endangered. Its conservation status is pessimistic, and the species requires immediate protection. Therefore, we supplemented a historical dataset to identify changes in this bird's range and population in northeast China over the long term. The study area spanned Heilongjiang Province, Jilin Province, and the northeast corner of Inner Mongolia in northeast China. We integrated an ecological niche model (BIOMOD2) with long‐term ecological data on this species to estimate the magnitude of change in distribution over time. Our results revealed a 35.25% reduction in the current distribution of this species compared to their potential distribution in the 1970s. This decline is expected to continue under climate change. For example, the future range loss was estimated to be 38.79 ± 0.22% (8.64–90.19%), and the actual state could be worse, because the baseline range of the model was greater than the real range in the 2000s, showing a 12.39% overestimation. To overcome this poor outlook, a conservation strategy should be established in sensitive areas, including the southwestern Greater Khingan Mountains and northern Lesser Khingan Mountains. Actions that should be considered include field investigations, establishing a monitor network, designing ecological corridors, and cooperating with local inhabitants, governments, and conservation biologists to improve the conservation of the black‐billed capercaillie.

## 1 INTRODUCTION

Knowledge of the ecological geographical characteristics for a species is the foundation to its biological conservation (Guisan et al., 2013; Valladares et al., 2014). However, such biogeographical processes can take decades or longer to run their course (Davies, Colombo, Hanley, & Thompson, 2014; Rick & Lockwood, 2012; Turvey, Crees, & Di Fonzo, 2015). Therefore, ecologists require an ecological baseline to understand the long‐term ecological responses of ecosystems to anthropogenic activities and climate change (Beaugrand, Edwards, Raybaud, Goberville, & Kirby, 2015; Fonzo, Collen, & Mace, 2013; Rick & Lockwood, 2012). Historical ecological data can be used to document species records over time (McClenachan, Ferretti, & Baum, 2012; Turvey et al., 2015; Yang et al., 2016) and help ecologists integrate future perspectives within historical contexts to provide unique insights into the long‐term dynamics of endangered species (Beaugrand et al., 2015; Scherrer et al., 2017). In fact, the application of long‐term ecological data, particularly historical data, is often hampered by data limitations, including incomplete and spatially biased datasets (Boakes et al., 2010; Hortal, Jimenez‐Valverde, Gomez, Lobo, & Baselga, 2008; McClenachan et al., 2012). Despite recognizing the considerable potential benefits of long‐term ecological archives to conservation research, policy, and practices, relatively few studies have analyzed multidecadal datasets or have examined changes in biogeography from a historical perspective (Davies et al., 2014; Li, Waller, & Syphard, 2017; McClenachan et al., 2012; Turvey et al., 2015). Therefore, in conservation efforts, there is a need to provide effective methods to assess the utility and potential limitations of long‐term ecological data to develop a meaningful understanding of population dynamics from the past to the future (McClenachan et al., 2012; Yang et al., 2016; Zhang et al., 2016, 2017).

Multiple resources and ecological niche model have proven to be effective methods of reconstructing historical distributions from long‐term ecological data (Zhang et al., 2017). Multiple cross‐checked data resources can reduce nonstandardized sampling and errors and provide sufficient data for further analysis (Boakes et al., 2010; Fonzo et al., 2013; Ren et al., 2016). Meanwhile, ecological niche modeling (ENM) is useful for constructing species distributions to support conservation efforts (Guisan et al., 2013; Luo, Jiang, & Tang, 2015; Veloz et al., 2015). Discrepancies between different species distribution models (SDMs) can be large, making the choice of the appropriate model difficult (Cuyckens et al., 2016; Elith & Leathwick, 2009; Elith et al., 2011; Renner & Warton, 2013; Veloz, 2009). In this context, ensemble forecasting approaches may be an appropriate choice (Thuiller, Lafourcade, Engler, & Araújo, 2009). BIOMOD is considered a suitable platform for ensemble forecasting of species distributions (Cianfrani et al., 2011; Thuiller et al., 2009). Combining multiple data resources with ecological niche modeling can be used to evaluate a species sensitively and comprehensively by revealing changes that occur over decades.

IUCN's assessment information of black‐billed capercaillie (*Tetrao urogalloides*) is Least Concern (LC) on the red list, its low conservation status is due to the wider distribution of the species in Siberia, and assessment report also clearly pointed out that the population may have declined due to over‐hunting in northeast China (http://www.iucnredlist.org/details/22679491/0) (González et al., 2012; Moss et al., 2014; Wang, Ren, He, & Zhu, 2008). *Tetrao urogalloides urogalloides* is one of three subspecies and is mainly distributed in northeast Asia, where northeast China is the southernmost distribution of this species, and it is a large forest‐dependent species associated with conifer‐dominated forests, and considered as a significant species for boreal and montane forests (He, Wan, Tian, & Ge, 2004; Ren et al., 2016; Sykes, Neiffer, Terrell, Powell, & Newton, 2013; Yin, Ge, Guan, Sun, & Li, 2009; Zhang, Ding, Ding, & Zheng, 2003). Previous research has implied that population declines have been driven by habitat destruction, with a 53% decrease in observation records between about 1950 and 2010 (Ren et al., 2016). Given that climatic regimes are expected to change further in coming years, as noted in the Paris Climate Agreement and by the Intergovernmental Panel on Climate Change (Huang, Yu, Dai, Wei, & Kang, 2017; IPCC, 2015), such changes may force animals to move toward higher elevations and latitudes, leading to habitat loss and fragmentation and range contraction. Therefore, the conservation situation is considered pessimistic for the black‐billed capercaillie in the near future. To support conservation efforts, we should strive to understand the changes in its range and associated driving factors based on long‐term dynamics that may not be available from short‐term ecological studies.

In this study, we integrated ENM's with long‐term ecological records of black‐billed capercaillie to estimate the magnitude of changes in their distribution over time. To this goal, we cross‐checked datasets from previous research and added new records collected from multiple resources (e.g., gazetteers and journal articles); reconstructed climate data to represent environmental variables, including historical climate data and general circulation models (GCMs); estimated the potential habitat for each period using BIOMOD2; and evaluated changes in the distribution range and possible impact factors. Our results offer an understanding of the ecological and biogeographic characteristics of the black‐billed capercaillie population decline over a long period of time, which can be used to improve the power of conservation management for the species and other species in similar ecologic niches.

## 2 MATERIALS AND METHODS

### 2.1 Study area

**Figure 1:**
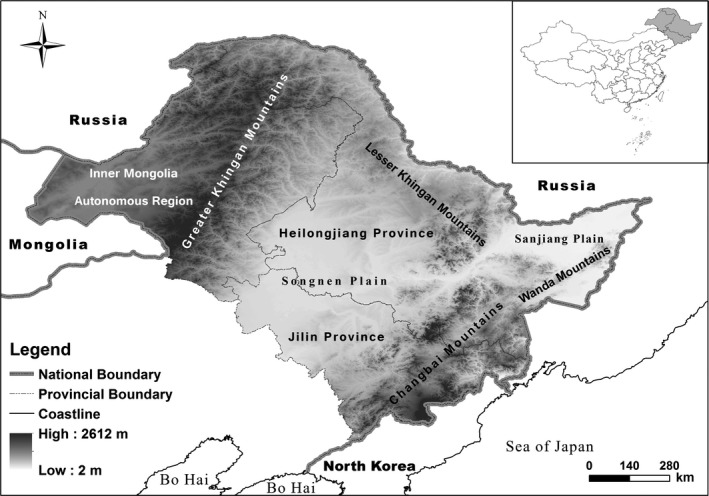
Study area in northeast China

The study area spanned Heilongjiang Province, Jilin Province, and the northeast corner of Inner Mongolia (N40.08°–53.28°, E115.50°–135.1°; the boundary of northeast China was obtained from the Thematic Database for Human‐Earth System [http://www.data.ac.cn/index.asp]), covering an area of about 938,000 km^2^ with an elevation range of 2–2,576 m (Figure 1) (Deng, Jiang, Zhan, He, & Lin, 2010). The region is characterized by a continental monsoon climate, with an annual precipitation of 400–1,000 mm and annual average temperature of 1–4°C. Vegetation flourishes, and tree cover is about 42.9% (Leng, He, Bu, & Hu, 2006). Coniferous forests are mainly located in the Greater Khingan Mountains and are dominated by *Larix gmelinii*. Broad‐leaved mixed forests appear in the Lesser Khingan Mountains, Changbai Mountains, and Wanda Mountains and are dominated by *L*.* gmelinii*,* Pinus koraiensis*,* Quercus mongolica*,* Tilia amurensis*, and *Betula platyphylla* (Deng et al., 2010; Xiaofeng et al., 2011; Yang & Xu, 2003). These complex ecological systems support many wildlife species, including the black‐billed capercaillie (*T. urogalloides*), black grouse (*Lyrurus tetrix*), and hazel grouse (*Bonasa bonasia*) (He et al., 2004; Yin et al., 2009; Zhang et al., 2003; Zhu & Liu, 1989).

### 2.2 Database construction

Following a data collection process described in our previous research (Yang et al., 2016), we collected historical data from five sources: gazetteers, fauna records, nature reserve scientific surveys, bird records, and scientific research (including articles and specimen records). Next, we recorded the locality information from all extracted records on Google Earth. Finally, all records were classified by decade (Table 1), yielding 377 records from the 1970s (1970–1979), 308 from the 1980s (1980–1989), 257 from the 1990s (1990–1999), and 229 from the 2000s (2000–2016). To eliminate potential bias caused by clustered occurrences, we removed duplicate records within the same cell (size ~1 km^2^). The total records selected and classified into different decades (Table 1) after this elimination included 376 records from the 1970s, 278 from the 1980s, 227 from the 1990s, and 199 from the 2000s. The records in the 2000s were selected to derive future climate projections and were considered the baseline records.

**Table 1:** Estimates of range sizes and accuracies of the species distribution models at different periods in northeast China

Period	Occurrence	Records used for modeling	TSS	Elevation	Range
1970s	377	376	0.646 ± 0.017	678.174	434,108
1980s	308	278	0.700 ± 0.008	694.481	339,087
1990s	257	227	0.765 ± 0.008	721.188	241,649
2000s	229	199	0.791 ± 0.007	717.995	266,770
Baseline	229	199	0.759 ± 0.015	704.079	315,928
2030RCP2.6	—	—	—	742.587	221,842
2030RCP4.5	—	—	—	717.879	209,024
2030RCP6.0	—	—	—	742.024	261,079
2030RCP8.5	—	—	—	716.071	194,724
2050RCP2.6	—	—	—	756.142	235,004
2050RCP4.5	—	—	—	737.877	219,465
2050RCP6.0	—	—	—	773.732	204,281
2050RCP8.5	—	—	—	823.983	116,180
2070RCP2.6	—	—	—	700.142	169,674
2070RCP4.5	—	—	—	747.481	121,458
2070RCP6.0	—	—	—	803.943	175,558
2070RCP8.5	—	—	—	815.224	30,979

Occurrence data were collected from five sources and verified (see Section 2—Data). Records were obtained from occurrence points, and duplicate records within the same cell were removed. All results were derived from the distribution models. Model accuracy was determined based on the TSS (mean ± *SD*). The ranges were determined as the total of the number of related pixels (~1 km^2^).

### 2.3 Environmental variable selection

Historical daily climate data were downloaded from the surface daily climate dataset (code: SURF_CLI_CHN_MUL_DAY, ver. 3.0, China Meteorological Data Service Center [http://data.cma.cn]), including mean temperature, maximum temperature, minimum temperature, and precipitation (recorded daily at 20:00). The climate variables for 12 months per 10 years were calculated by interpolation of complex multivariate data using thin plate smoothing splines with ANUSPLIN ver. 4.36. For each period, bioclimatic variables were derived from the monthly temperature and rainfall values with the dismo package in R (R Development Core Team, Vienna, Austria). From this, we obtained 19 history bioclimatic variables (Appendix S1).

To derive future climate projections, we used GCMs based on four representative concentration pathways (RCPs) to represent future climate change based on the IPCC Assessment Report (IPCC 2015). The 19 bioclimatic variables for the current conditions (baseline) (i.e., interpolations of observed data representative of 1960–1990) were downloaded from WorldClim ver. 1.4 (http://www.worldclim.org/) (Hijmans, Cameron, Parra, Jones, & Jarvis, 2005). Then, we downloaded bioclimatic variables (MIROC5; Atmosphere and Ocean Research Institute [The University of Tokyo], National Institute for Environmental Studies, and Japan Agency for Marine‐Earth Science and Technology) for the 2030s, 2050s, and 2070s from the CGIAR Research Program on Climate Change, Agriculture and Food Security (http://www.ccafs-climate.org/) (Appendix S1).

Topography data were obtained from the SRTM 90 m Digital Elevation Database (http://srtm.csi.cgiar.org/), which were used to calculate slope and aspect data. In total, 22 variables were obtained for further research (Appendix S1). All ecological data were obtained in a raster structure with a cell size of 1 km^2^.

To reduce collinearities among the variables used for modeling and avoid inappropriately complex models, we built a Spearman correlation matrix for all variables (Ranjitkar et al., 2016; Zhang et al., 2017). We drew a tree diagram (Appendix S1) using the Hmisc package in R to facilitate variable selection. In the tree diagram, correlated variables were linked by lines and clustered together. For any coefficient >0.70, we removed the variable with a lower value in the percentage importance as calculated by Maxent3.3.3k (Elith et al., 2011; Phillips, Anderson, & Schapire, 2006). Finally, each historical period had special variables for ecological niche modeling, and current conditions (baseline) had special variables for ensemble forecasting of the species distribution.

### 2.4 Species distribution modeling analyses

We estimated the potential distribution of the black‐billed capercaillie in northeast China over time using BIOMOD2. Implemented in the BIOMOD R package (version 3.3–7) (Phillips et al., 2006; Thuiller et al., 2009), we used 10 modeling techniques: generalized linear modeling (GLM), generalized additive modeling (GAM), generalized boosting modeling (GBM), classification tree analysis (CTA), artificial neural network (ANN), flexible discriminant analysis (FDA), surface range envelop (SRE/BIOCLIM), multivariate adaptive regression splines (MARS), random forest (RF), and maximum entropy (MAXENT. Phillips). All techniques were applied to presence data for each period and to the pseudo‐absences for the models developed in BIOMOD. For each period, each SDM was evaluated measuring the true skill statistic (TSS) (Allouche, Tsoar, & Kadmon, 2006), with five evaluation runs. For the TSS, 50 modeling evaluation results were obtained, and the average of the TSS was set as the threshold for building the ensemble models. Then, the potential distribution was calculated from an ensemble set of models or predictions. To reduce the uncertainty in our research, we repeated the models five times, yielding a total 85 SDMs: 20 SDMs from the historical climate (including 1970s, 1980s, 1990s, and 2000s), five SDMs under current conditions (baseline), and 60 SDMs for MIROC5, with four RCPs in each period (including the 2030s, 2050s, and 2070s). The final logistic results for each period were weighted based on the TSS. To transform the models from environmental suitability into presence–absence distributions, we used the threshold (*P*) calculated with the weighted average cutoff calculated by BIOMOD. From this, all outputs were divided into two groups; outputs above the threshold (>*P*) were grouped as “present,” while all other values (<*P*) were considered “absent” (see details in Appendix S2).

It is important to quantify the spatial pattern of changes in distributions. Therefore, the predicted current range/historical range (CR), potential range loss (RL), and potential range gain (RG) were assessed by summing the numbers of the respective related pixels. Then, the range turnover (RT) ([RG + RL]/[CR + RG]) was calculated (Luo et al., 2015). The change in range was calculated with BiodiversityR (Ranjitkar et al., 2016). The elevation and center of the distribution in different periods were extracted. Spatial analyses were performed using ArcGIS (ver. 10.2; ESRI Inc., Redlands, CA, USA), Excel 2013 (Microsoft Corp., Redmond, WA, USA), R software (R Development Core Team, Vienna, Austria), and SPSS for Windows (ver. 20.0; SPSS Inc., Chicago, IL, USA).

## 3 RESULTS

### 3.1 Database construction results

A total of 358 records from previous research (Yang et al., 2016), 75 from articles and local historical documents (He et al., 2004; Yin et al., 2009; Zhang et al., 2003; Zhao, 2001), five from bird records, four from specimens, and two from nature reserve scientific surveys were collected. Conflicting records with unsubstantiated metadata, such as those lacking relevant or detailed descriptions, were excluded from the analysis.

### 3.2 Model performance

The number of available records of the black‐billed capercaillie in each decade ranged from 199 to 376 (Table 1). The average TSS for the consensus model was 0.732 ± 0.052 (Table 1). The period of the 2000s (2000–2016) had the highest TSS among all periods, while the 1950s had the lowest.

### 3.3 Range change in the past

**Figure 2:**
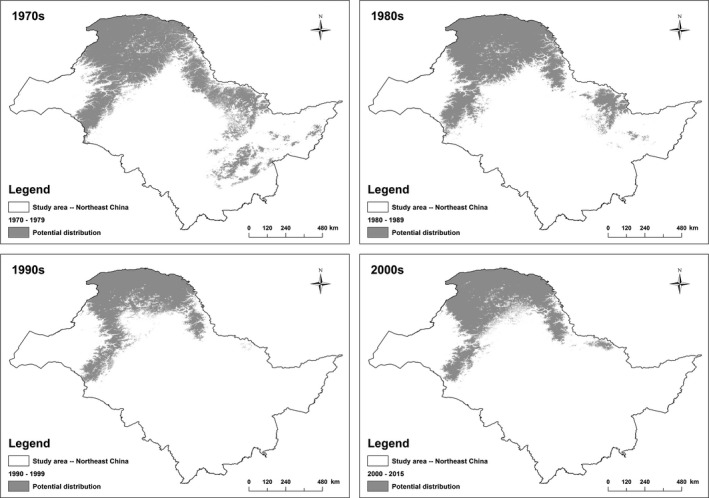
Historical potential distribution of the black‐billed capercaillie in different decades

The black‐billed capercaillie was widely distributed in all four areas of northeast China, including the Greater Khingan Mountains, Lesser Khingan Mountains, Changbai Mountains, and Wanda Mountains (Figure 2). In the 1970s, their potential distribution area was 46.28% of the total study area (938,000 km^2^). Compared to the potential distribution in the 1970s, the range decreased by 21.89% and 44.33% in the 1980s and 1990s, respectively. The elevation increased compared to the 1970s, with an increase of 2.40% and 6.34% in the 1980s and 1990s, respectively (see Appendix S3). There may have been regional extinctions in the Changbai and Wanda Mountains in the late 1980s, in agreement with previous research (Ren et al., 2016). In the 2000s (2000–2016), the distribution seemed to have extended, with an increase of about 10% compared to the 1990s. However, the outlook was still poor. The distribution became concentrated in the core areas of the Greater Khingan Mountains and northern Lesser Khingan Mountains, with an increase in elevation and range shift to the northwest (Figure 2; see details in Appendix S3).

Overall, compared to the 1970s, RL and RG for all periods (1980s, 1990s, and 2000s) were 30.36 ± 0.12% (14.21–41.62%) and 4.57 ± 0.02% (2.72–7.68%), respectively. RT was 37.93 ± 0.08% (27.46–45.81%) (see Appendix S3).

### 3.4 Range changes under climate change

**Figure 3:**
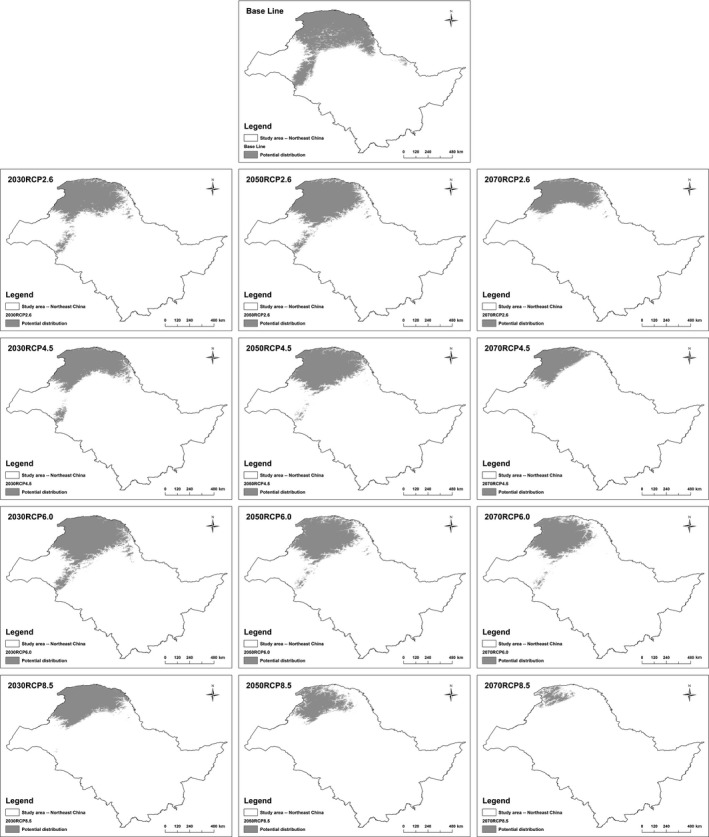
Potential distribution of the black‐billed capercaillie under climate change

Under current conditions (baseline), black‐billed capercaillie was mainly distributed in the Greater Khingan Mountains and northern Lesser Khingan Mountains (Figure 3). Their potential distribution area was 33.68% of the total study. Compared to the baseline, RL and RG for all scenarios were 38.79 ± 0.22% (8.64–90.19%) and 4.26 ± 0.03% (0.01–8.76%), respectively. RT was 45.77 ± 0.18% (23.99–90.20%) (see Appendix S3).

Range loss significantly increased in future projections (24.18 ± 0.09% [8.64–33.79%] in the 2030s; 33.42 ± 0.17% [16.85–62.22%] in the 2050s; 58.76 ± 0.20% [40.02–90.19%] in the 2070s), and the RG decreased (5.65 ± 0.02% [4.05–8.72%] in the 2030s; 5.26 ± 0.03% [1.01–8.76%] in the 2050s; 1.86 ± 0.02% [0.01–4.41%] in the 2070s). In addition, the RT increased (33.68 ± 0.06% [23.99–41.06%] in the 2030s; 42.05 ± 0.13% [31.61–63.59%] in the 2050s; 61.57 ± 0.18% [46.78–90.20%] in the 2070s) (see Appendix S3). Overall, the distribution tended to decrease dramatically, with the center shifting from southeast to northwest and the elevation increasing in the near future (see details in Appendix S3).

To offer a future perspective with a historical context, the baseline was compared to the 2000s. An overestimation of area in the baseline was identified, mainly located in the eastern Greater Khingan Mountains, while an area of underestimation was mainly located in northern Lesser Khingan Mountains and western Greater Khingan Mountains. Compared to the 2000s, there was a 6.76% underestimation and 19.15% overestimation in the baseline. Overall, the baseline range was 12.39% larger than that in the 2000s (see details in Appendix S4). Therefore, the actual decrease under climate change may be larger than estimated.

## 4 DISCUSSION

Our investigation supports the use of long‐term ecological data to understand the dynamics of species responses to human pressures and climate change. The analyses controlled or tested for multiple issues affecting data quality, resolution, incompleteness, and biases that have not been addressed in previous studies, including the creation of a long‐term ecological dataset of the black‐billed capercaillie from multiple cross‐checked resources, construction of spatiotemporal geographical changes with species distribution models, and integration of changes in the near future with a historical context. These methods can be used to track range changes across longer timescales than those usually addressed in ecology or conservation biology. We suggest that future research addressing climate change should consider the differences between the baseline and more realistic current conditions (i.e., the 2000s in this study).

### 4.1 Model limitations

Our research contained several limitations, including common limitations associated with SDMs, such as data accuracy, variable representativeness, and spatiotemporal bias (Beck, Böller, Erhardt, & Schwanghart, 2014; Boakes et al., 2010; Ranjitkar et al., 2016; Rowe et al., 2014). In addition, integrating the future changes within a historical context was only based on the difference between the range from the baseline and the 2010s, because the GCMs were calculated from the baseline (Hijmans et al., 2005). However, historical records of black‐billed capercaillie presence already contain potential information on human influences and habitat information, which could reduce bias caused by missing predictors or variables representative of the model. The difference between the range from the baseline and the 2010s also offers an opportunity for qualitative analysis and provides a more realistic assessment under climate change.

### 4.2 Threats and conservation

Previous research has shown that anthropogenic influences, including deforestation, agricultural expansion, and overexploitation of terrestrial ecosystems, have occurred throughout northeast China in the past decades, leading to distribution declines (Moss et al., 2014; Ren et al., 2016). However, this influence is difficult to quantify and analyze due to the availability of historical data on human activities (e.g., land cover and vegetation cover). Changes in human population size can be used as an indicator to reflect the scale of urbanization, deforestation, and reclamation of farmland. Therefore, data on human population size within the potential distribution of the black‐billed capercaillie were collected to help visualize this relationship (see Appendix S4).

Human population density has increased in southeastern northeast China since the 1970s. An increase in the Lesser Khingan Mountains and Changbai Mountains has led to deforestation and habitat destruction. Several studies have suggested that the human population center in northeast China has moved from the northeast to southwest along with the concentration of urban areas, and this trend is expected to continue in the near future (Guo, Li, Chen, & Gan, 2015; Jia & Gu, 2016). By contrast, the distribution center of the black‐billed capercaillie is showing a northern shift.

Our results suggest that human‐associated habitat destruction has been the main driver of range loss in the species in the recent past, but climate change could become a prominent, if not leading, cause of range change in the coming decades. The Natural Forests Protection Program, which began in 1998, has yielded substantial improvements in the quantity and quality of forests in northeast China (Wei et al., 2014). Moreover, recent research has provided evidence that black‐billed capercaillie populations are recovering in several areas in the Greater Khingan Mountains, although populations elsewhere are still deceasing (He et al., 2004; Yin et al., 2009; Zhang et al., 2003). This is in agreement with our results of a range increase in the 2000s compared to the 1990s. Environmental protection programs and population trends (i.e., southern shift and urbanization) suggest that anthropogenic influences may not dramatically increase in the near future, and that the range change will be mainly driven by climate change.

China has established nature reserves network in our study area, especially Zhongyangzhan Nature Reserve for black‐billed capercaillie, but most of population are not distributed in nature reserves. According to our results, the state of conservation is still critical, and we suggest several actions that should be considered to improve the situation.

Field investigations should be performed in the southwestern Greater Khingan Mountains and northern Lesser Khingan Mountains, because the populations in these areas may suffer from climate change. In addition, studies on habitat selection, reproductive biology, and interactions between populations in these areas are vital to improve our understanding of the response to climate change. A monitoring network associated with protected areas should be established, particularly in areas sensitive to climate change. Protection efforts of the black‐billed capercaillie should be coordinated with local inhabitants to promote ecological tourism and conservation‐related enterprises that increase income for local inhabitants. This could change the lifestyle of locals, reducing disturbances to black‐billed capercaillie habitat in mountainous areas. In addition, community co‐management should be established with the cooperation of local inhabitants, governments, and conservation biologists. Ecological corridors should be designed to maximize habitat connectivity. Meanwhile, to facilitate acceptance by local communities whose lands are affected by such conservation networks, an incentive program should be launched to encourage locals to take part in conservation efforts.

## CONFLICT OF INTEREST

None declared.

## AUTHOR CONTRIBUTIONS

All authors designed the experiment. Li Yang and Xiaofeng Luan performed the data collection, the structure of manuscript, and drafted the first version of the manuscript. Chao Zhang performed the data collection, experiments, and data analysis. Minhao Chen performed data collection and interpretation. Jingxin Li and Lei Yang worked in the data analysis. Zhaomin Huo and Shahid Ahmad worked in the data collection. All co‐authors participated in the scientific discussions and commented on the manuscript.
