# Supplementary material for: Long‐term ecological data for conservation: Range change in the black‐billed capercaillie (Tetrao urogalloides) in northeast China (1970s–2070s)
Source: Ecol Evol. 2018 Mar 23;8(8):3862–70. doi: 10.1002/ece3.3859 (PMC5916277; doi:10.1002/ece3.3859)
Supplement: Supplementary file 1 [file ECE3-8-3862-s001.docx]

**APPENDIX A**

A. Tree diagram with eco-geographic variables to facilitate variable selection

B. The percent contribution and permutation importance of variables in different periods

C. Eco-geographic variables used in species distribution models of Black-billed Capercaillie in Northeast China for different periods.

**A. Tree diagram with eco-geographic variables to facilitate variable selection**


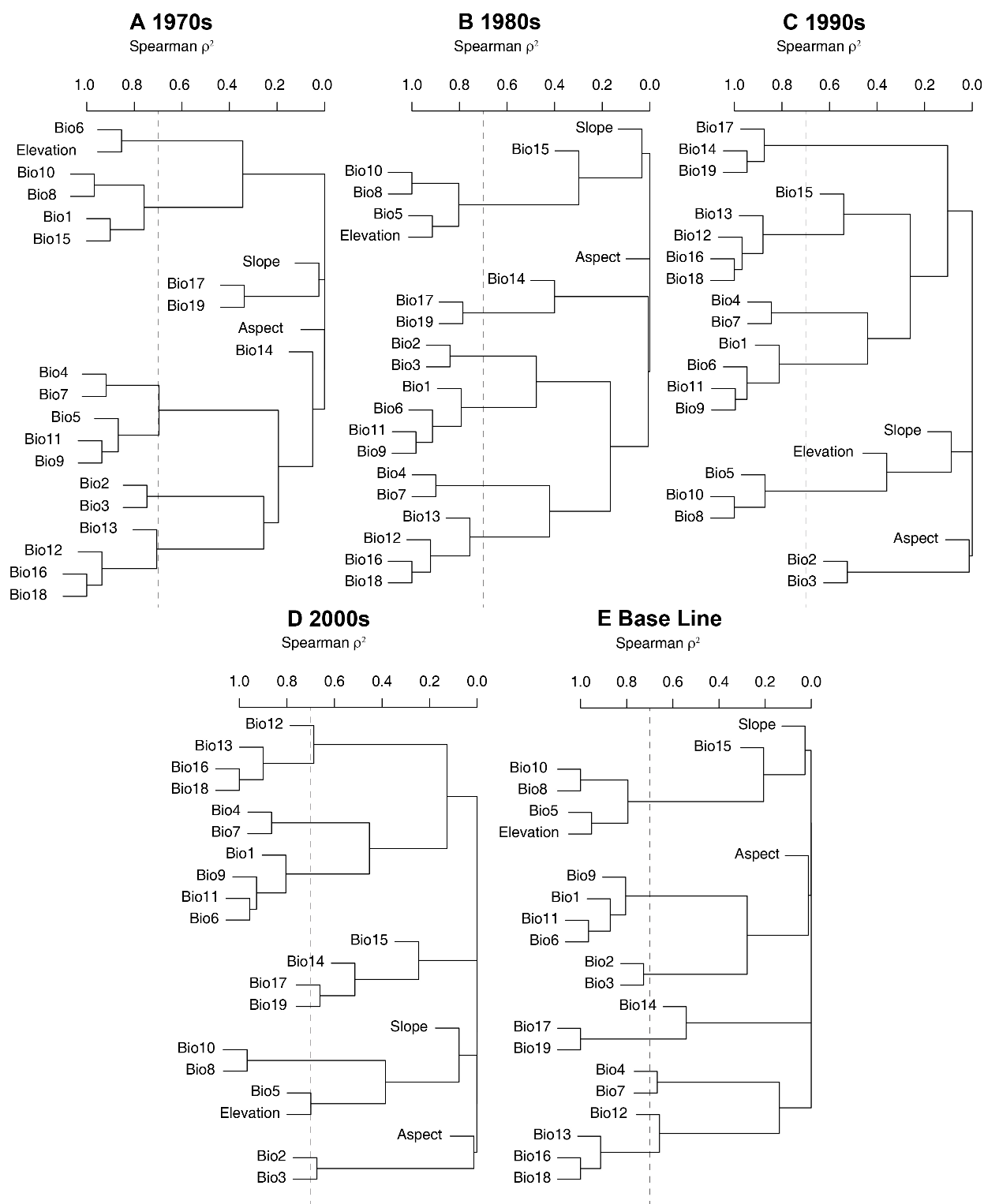


**Fig. S1. Tree diagram with eco-geographic variables to facilitate variable selection**

**Note: Dash line means coefficient = 0.70**

**B. The percent contribution and permutation importance of variables in different periods**

| **Periods** | **Variable** | Bio1 | Bio2 | Bio3 | Bio4 | Bio5 | Bio6 | Bio7 | Bio8 | Bio9 | Bio10 | Bio11 | Bio12 | Bio13 | Bio14 | Bio15 | Bio16 | Bio17 | Bio18 | Bio19 | ELE | ASP | SLO |
| --- | --- | --- | --- | --- | --- | --- | --- | --- | --- | --- | --- | --- | --- | --- | --- | --- | --- | --- | --- | --- | --- | --- | --- |
| **1970s** | **Percent contribution** | 9.2 | 4.6 | 1.7 | 1.7 | 0.3 | 27.9 | 1.1 | 2.1 | 10.2 | 20.9 | 0.6 | 1.4 | 2.2 | 0.6 | 1.7 | 0.2 | 0.7 | 0.0 | 3.1 | 2.0 | 0.8 | 7.3 |
|  | **Permutation importance** | 2.7 | 3.0 | 3.0 | 2.2 | 1.3 | 3.1 | 0.4 | 10.9 | 7.0 | 29.1 | 0.2 | 0.6 | 4.2 | 0.6 | 12.3 | 0.1 | 3.6 | 0.0 | 5.3 | 5.9 | 0.6 | 3.9 |
| **1980s** | **Percent contribution** | 35.2 | 4.9 | 0.9 | 0.4 | 29.2 | 1.0 | 1.1 | 0.6 | 4.3 | 2.5 | 1.5 | 0.2 | 1.3 | 1.3 | 1.8 | 0.2 | 5.6 | 0.0 | 3.5 | 1.1 | 1.3 | 2.5 |
|  | **Permutation importance** | 21.9 | 6.2 | 0.6 | 1.5 | 12.9 | 5.8 | 0.3 | 2.6 | 2.8 | 12.3 | 2.4 | 0.1 | 1.4 | 1.2 | 4.0 | 0.7 | 6.5 | 0.1 | 1.0 | 10.7 | 1.4 | 3.5 |
| **1990s** | **Percent contribution** | 30.4 | 3.0 | 1.3 | 0.8 | 0.3 | 1.3 | 1.6 | 0.5 | 2.8 | 39.8 | 3.1 | 0.3 | 0.3 | 4.5 | 1.5 | 0.5 | 4.5 | 0.3 | 1.2 | 0.3 | 1.0 | 0.8 |
|  | **Permutation importance** | 21.3 | 3.3 | 4.6 | 1.5 | 1.1 | 3.8 | 1.6 | 0.8 | 6.9 | 28.6 | 2.5 | 0.8 | 0.8 | 3.8 | 3.4 | 2.7 | 5.2 | 1.2 | 1.3 | 1.7 | 2.3 | 0.7 |
| **2000s** | **Percent contribution** | 35.4 | 3.4 | 0.6 | 0.1 | 1.6 | 1.1 | 0.4 | 0.1 | 1.9 | 37.8 | 4.6 | 2.7 | 0.4 | 0.5 | 1.0 | 0.9 | 1.2 | 0.2 | 2.5 | 0.7 | 1.0 | 1.8 |
|  | **Permutation importance** | 27.9 | 9.3 | 1.0 | 0.2 | 1.8 | 1.5 | 0.6 | 0.2 | 0.3 | 20.2 | 11.5 | 1.8 | 0.2 | 2.5 | 1.6 | 1.7 | 4.2 | 0.4 | 1.5 | 4.8 | 2 | 4.7 |
| **Base Line** | **Percent contribution** | 45.5 | 1.9 | 0.4 | 3.9 | 2.7 | 2.9 | 3.5 | 0.8 | 25 | 2.7 | 2.7 | 1.3 | 2.0 | 0.0 | 1.2 | 0.3 | 0.4 | 0.0 | 0.0 | 0.3 | 1.1 | 1.2 |
|  | **Permutation importance** | 21.9 | 9.7 | 0.5 | 1.8 | 2.2 | 2.0 | 5.6 | 2.0 | 3.2 | 11.5 | 5.3 | 3.1 | 10.8 | 0.0 | 3.0 | 0.5 | 5.4 | 0.3 | 0.3 | 3.3 | 2.2 | 5.5 |

Table S1 The percent contribution and permutation importance of variables in different periods

C. **Eco-geographic variables used in species distribution models of Black-billed Capercaillie in Northeast China for different periods**

TableS2 Eco-geographic variables used in species distribution models of Black-billed Capercaillie in Northeast China for different periods.

| Variables | Describe | Code | 1970s | 1980s | 1990s | 2000s | Baseline |
| --- | --- | --- | --- | --- | --- | --- | --- |
| BIO1 | Annual Mean Temperature | BIO1 |  | ● | ● | ● | ● |
| BIO2 | Mean Diurnal Range (Mean of monthly (max temp - min temp)) | BIO2 |  | ● | ● | ● | ● |
| BIO3 | Isothermality (BIO2/BIO7) (* 100) | BIO3 | ● |  | ● | ● |  |
| BIO4 | Temperature Seasonality (standard deviation *100) | BIO4 | ● | ● |  |  | ● |
| BIO5 | Max Temperature of Warmest Month | BIO5 |  | ● |  | ● |  |
| BIO6 | Min Temperature of Coldest Month | BIO6 |  |  |  |  |  |
| BIO7 | Temperature Annual Range (BIO5-BIO6) | BIO7 |  |  | ● | ● | ● |
| BIO8 | Mean Temperature of Wettest Quarter | BIO8 |  |  |  |  |  |
| BIO9 | Mean Temperature of Driest Quarter | BIO9 | ● |  |  |  |  |
| BIO10 | Mean Temperature of Warmest Quarter | BIO10 | ● |  | ● | ● | ● |
| BIO11 | Mean Temperature of Coldest Quarter | BIO11 |  |  |  |  |  |
| BIO12 | Annual Precipitation | BIO12 |  |  |  | ● | ● |
| BIO13 | Precipitation of Wettest Month | BIO13 | ● | ● |  |  | ● |
| BIO14 | Precipitation of Driest Month | BIO14 | ● | ● |  | ● | ● |
| BIO15 | Precipitation Seasonality (Coefficient of Variation) | BIO15 |  | ● | ● | ● | ● |
| BIO16 | Precipitation of Wettest Quarter | BIO16 |  |  | ● | ● |  |
| BIO17 | Precipitation of Driest Quarter | BIO17 | ● | ● | ● | ● | ● |
| BIO18 | Precipitation of Warmest Quarter | BIO18 |  |  |  |  |  |
| BIO19 | Precipitation of Coldest Quarter | BIO19 | ● |  |  |  |  |
| Elevation |  | ELE | ● |  | ● | ● |  |
| Aspect |  | ASP | ● | ● | ● | ● | ● |
| Slope |  | SLO | ● | ● | ● | ● | ● |

Note: History climate data can be downloaded from China Meteorological Data Service Center (<http://data.cma.cn/>);

Current climate data can be downloaded from WorldClim-Global Climate Data (version1.4) (<http://www.worldclim.org/>);

Future climate variables can be downloaded from Agriculture and Food Security (MIROC5) (<http://www.ccafs-climate.org/>);

Topography data were obtained from the SRTM 90m Digital Elevation Model v4.1 (<http://www.cgiar-csi.org/>);

The aspect and slope data were calculated from the topography data by Arcgis10.2.
