# Supplementary material for: Long‐term ecological data for conservation: Range change in the black‐billed capercaillie (Tetrao urogalloides) in northeast China (1970s–2070s)
Source: Ecol Evol. 2018 Mar 23;8(8):3862–70. doi: 10.1002/ece3.3859 (PMC5916277; doi:10.1002/ece3.3859)
Supplement: Supplementary file 2 [file ECE3-8-3862-s002.docx]

**APPENDIX B**

A. Approach for modeling species potential distributions

B. Approach for modeling species potential distributions in BIOMOD2

**A. Approach for modeling species potential distributions**

**
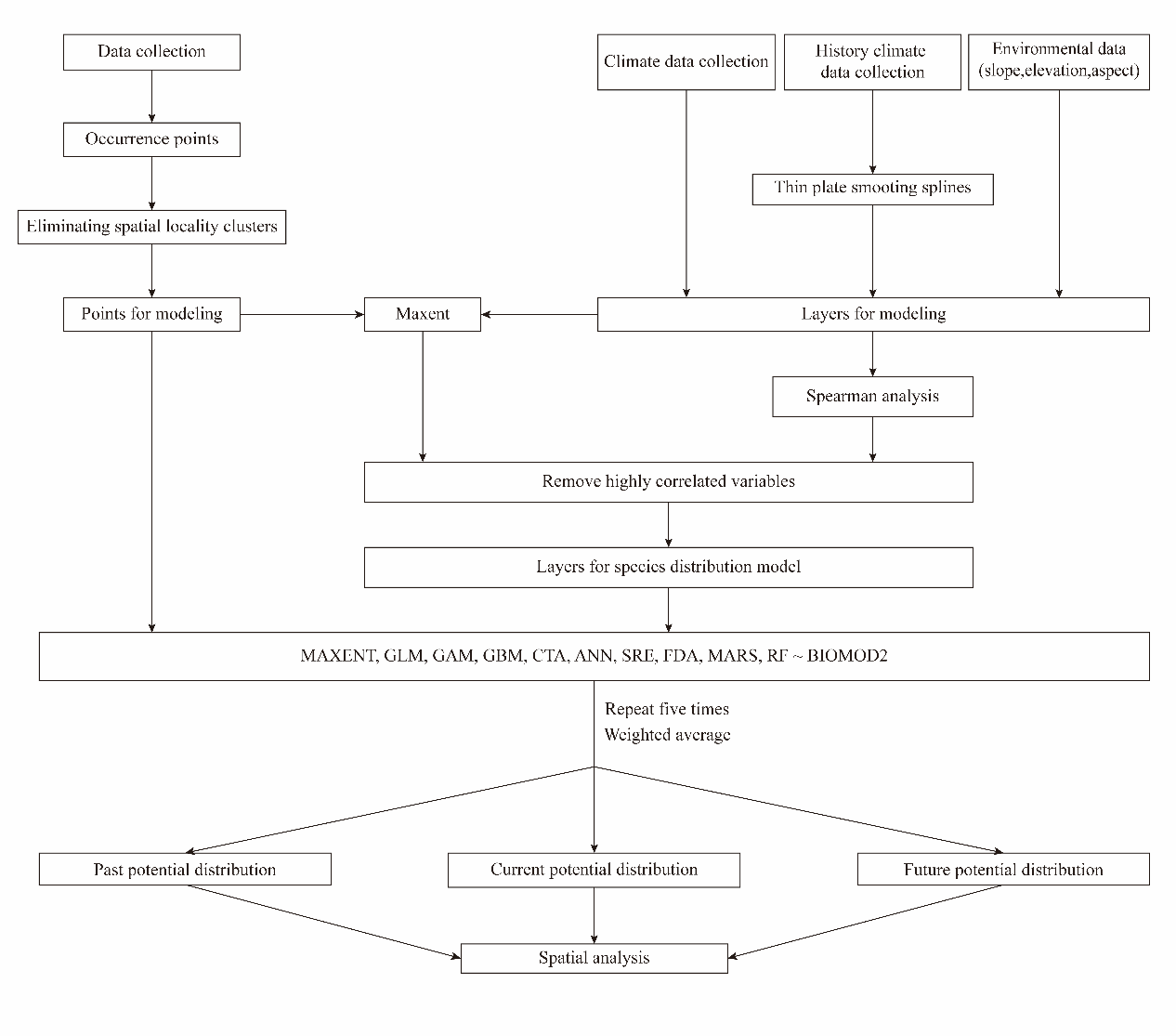
**

**Fig. S2** **Approach for modeling species potential distribution**

**B. Approach for modeling species potential distributions in BIOMOD2**


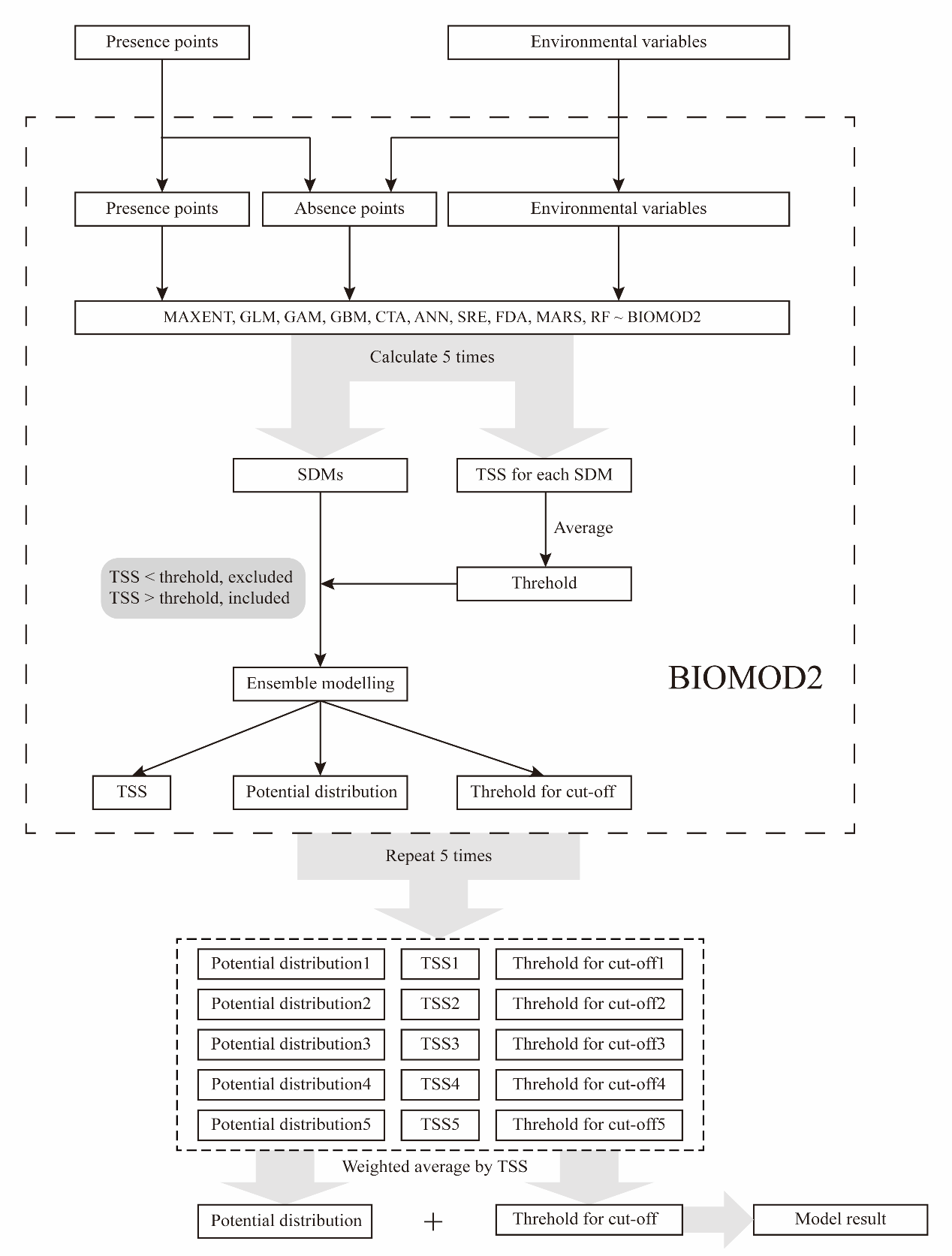


**Fig. S3** **Approach for modeling species potential distributions in BIOMOD2**
