# Supplementary material for: Long‐term ecological data for conservation: Range change in the black‐billed capercaillie (Tetrao urogalloides) in northeast China (1970s–2070s)
Source: Ecol Evol. 2018 Mar 23;8(8):3862–70. doi: 10.1002/ece3.3859 (PMC5916277; doi:10.1002/ece3.3859)
Supplement: Supplementary file 3 [file ECE3-8-3862-s003.docx]

**Appendix C**

A. The potential distribution in different periods compared with the 1970s (km^2^)

B. The potential distribution in different periods compared with the Base Line (km^2^)

C. Historical distribution center (°) and elevation (m) in different decades

D. Distribution center (°) and elevation (m) trends under climate change

E. Trend of the distribution center (°) and elevation (m) for the past and the future

1. **The potential distribution in different periods compared with the 1970s (km2)**

Table S3 The potential distribution in different periods compared with the 1970s (km^2^)

| Periods | always suitable | no longer suitable | new habitat |
| --- | --- | --- | --- |
| 1970s | 434108 | NA | NA |
| 1980s | 339087 | 95021 | 33353 |
| 1990s | 241649 | 192459 | 11797 |
| 2000s | 266770 | 167338 | 14324 |

**B. The potential distribution in different periods compared with the Base Line (km2)**

Table S4 The potential distribution in different periods compared with the Base Line (km^2^)

| Periods | always suitable | no longer suitable | new habitat |
| --- | --- | --- | --- |
| Base Line | 315928 | NA | NA |
| 2030RCP2.6 | 221842 | 94086 | 12788 |
| 2030RCP4.5 | 209024 | 106904 | 16646 |
| 2030RCP6.0 | 261079 | 54849 | 27553 |
| 2030RCP8.5 | 194724 | 121204 | 14449 |
| 2050RCP2.6 | 235004 | 80924 | 27681 |
| 2050RCP4.5 | 219465 | 96463 | 17212 |
| 2050RCP6.0 | 204281 | 111647 | 18376 |
| 2050RCP8.5 | 116180 | 199748 | 3179 |
| 2070RCP2.6 | 169674 | 146254 | 6428 |
| 2070RCP4.5 | 121458 | 194470 | 3081 |
| 2070RCP6.0 | 175558 | 140370 | 13941 |
| 2070RCP8.5 | 30979 | 284949 | 27 |

**C. Historical distribution center (°) and elevation (m) in different decades**





**Fig. S4** **Historical distribution center (°) and elevation (m) in different decades**

**D. Distribution center (°) and elevation (m) trends under climate change**





**Fig. S5** **Distribution center (°) and elevation (m) trends under climate change**

**E. Trend of the distribution center (°) and elevation (m) for the past and the future**


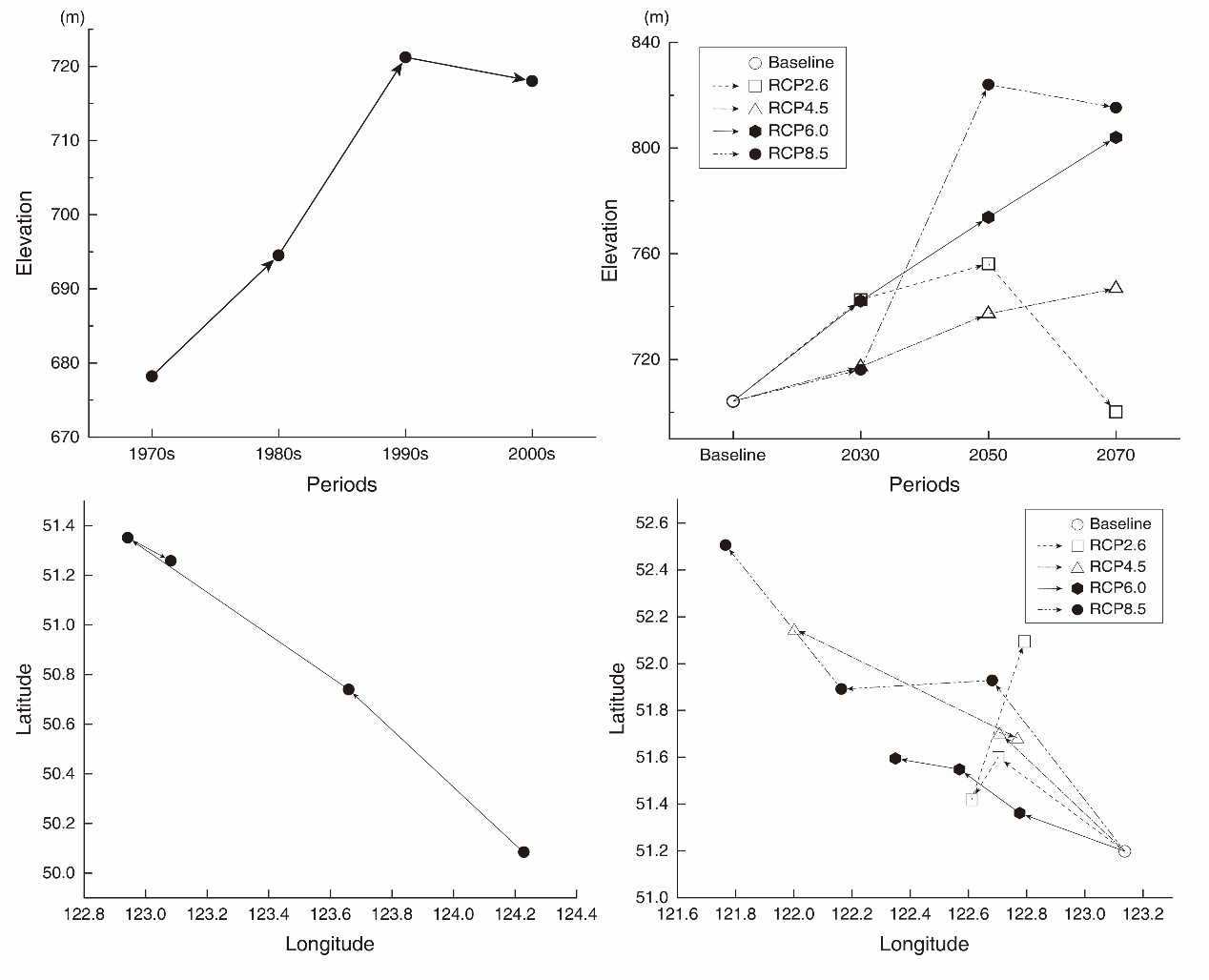


**Fig. S6 Trend of the distribution center (°) and elevation (m) for the past and the future**
