# Supplementary material for: Long‐term ecological data for conservation: Range change in the black‐billed capercaillie (Tetrao urogalloides) in northeast China (1970s–2070s)
Source: Ecol Evol. 2018 Mar 23;8(8):3862–70. doi: 10.1002/ece3.3859 (PMC5916277; doi:10.1002/ece3.3859)
Supplement: Supplementary file 4 [file ECE3-8-3862-s004.docx]

**Appendix D**

A. Differences in distribution area between the 2000s and the baseline

B. Human population density in northern China in different decades

**A. Differences in distribution area between the 2000s and the baseline**


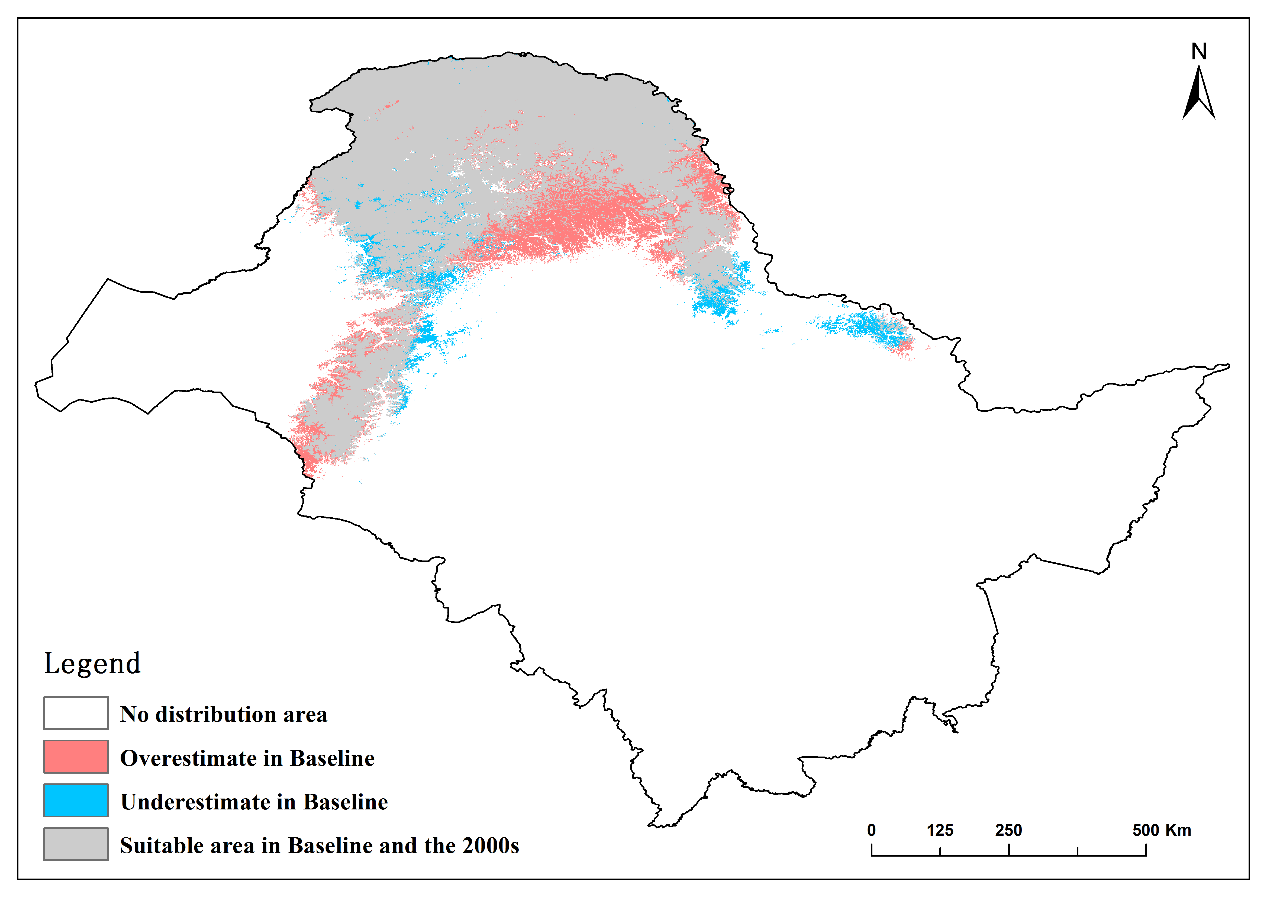


**Fig. S7 Differences in distribution area between the 2000s and the baseline**

**B. Human population density in northern China in different decades**


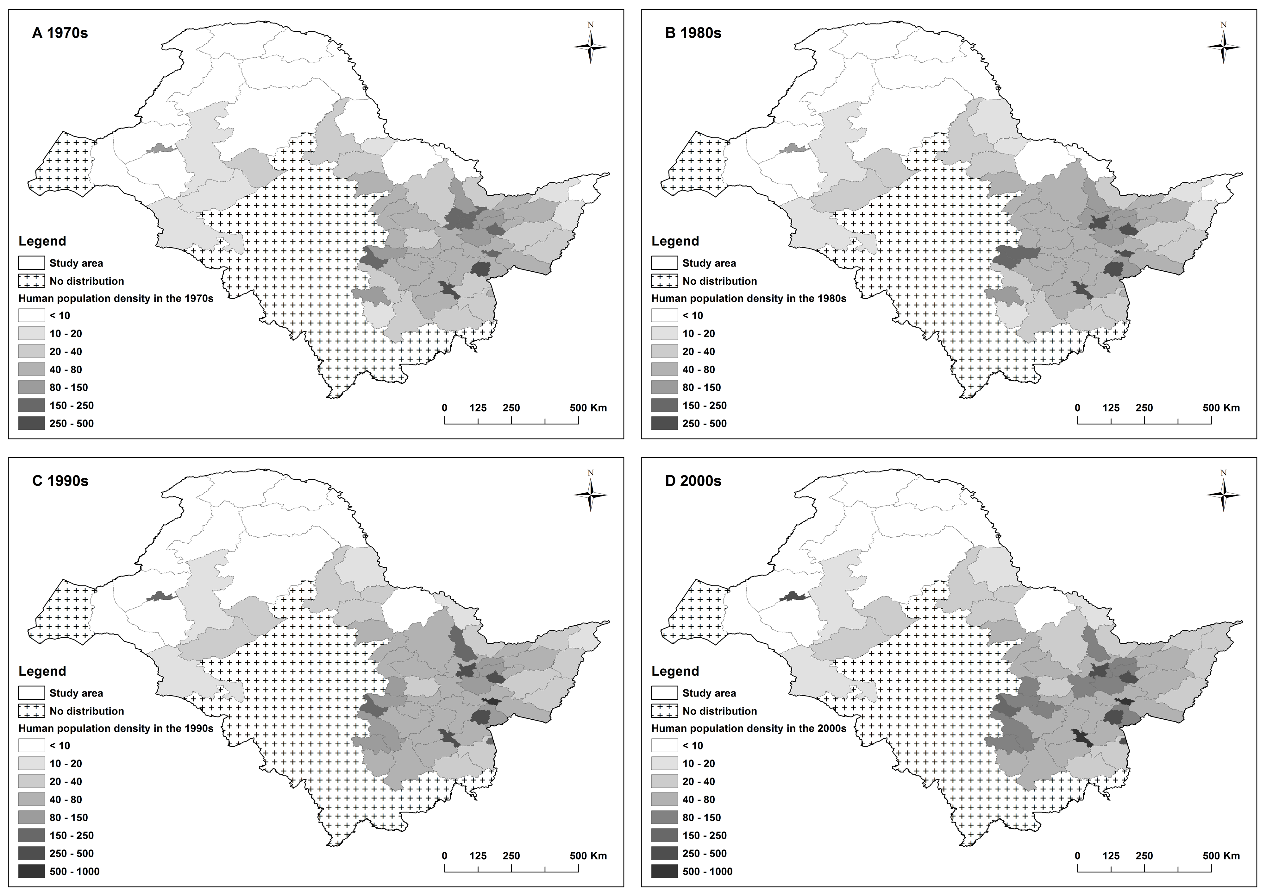


**Fig. S8 Human population density in northern China in different decades**
